# Development of Swine’s Digestive Tract Microbiota and Its Relation to Production Indices—A Review

**DOI:** 10.3390/ani10030527

**Published:** 2020-03-21

**Authors:** Damian Knecht, Paulina Cholewińska, Anna Jankowska-Mąkosa, Katarzyna Czyż

**Affiliations:** Institute of Animal Breeding, Faculty of Biology and Animal Sciences, Wroclaw University of Environmental and Life Sciences, Chelmonskiego 38C, 51-630 Wroclaw, Poland; damian.knecht@upwr.edu.pl (D.K.); anna.jankowska-makosa@upwr.edu.pl (A.J.-M.); katarzyna.czyz@upwr.edu.pl (K.C.)

**Keywords:** pigs, piglets, microbiota, gastrointestinal tract

## Abstract

**Simple Summary:**

Proper cooperation between digestive system microbiota and the host is an important issue in maintaining proper health condition, and—in the case of farm animals—production indices. In the case of pigs, microbiota significantly affect production parameters such as meat quality, growth rate or improvement of immune response to infections. Understanding of pig digestive system microbiota and factors affecting this is an important issue. This may enable improvement of animal performance and stabilization of microbiota during their growth, reducing the risk of metabolic or systemic diseases.

**Abstract:**

The development of research methods and tools related to microbiome investigation, as well as widened knowledge and awareness concerning the significance of microorganisms inhabiting mammalian organisms, has led to an increasing popularity of studies in this field. This review paper presents some issues related to the swine microbiome, its development starting from an early age of life and its status in adult animals, as well as factors affecting the microbiome in pigs. Attention is paid to the role of probiotics and prebiotics as alternatives to antibiotics in the context of post-weaning diarrhea treatment, and to the role of microorganisms inhabiting the digestive tract of pigs in performance indices formation. In veterinary and pork production practice, understanding of the swine microbiome and its relationships with the host organism may be useful in the prevention of some diseases and also in improvement of performance results of animals.

## 1. Introduction

The relationship between the host and the microorganisms that colonize it has been studied since the 1960s, mainly in humans. However, due to the development of research methods in the field of microbiology and molecular biology, knowledge related to its operation has been expanding over recent years. Microorganisms act as a specific ecosystem, which is adapted to the habitat and physiological status of the host. Populations of microorganisms include saprophytic microorganisms, commensals and incidentally pathogenic microorganisms [1,2,3].

In the case of pigs and other farm animals, it is important to know the interactions of gastrointestinal microbiota, since it is a significant issue for animal health status and production parameters such as meat quality, body weight as well as an enhanced immune response to porcine reproductive and respiratory syndrome (PRRSV) infection, as has been demonstrated in recent research on swine microbiota [4,5,6,7].

Due to the high degree of similarity in anatomy, physiology, immunology and brain growth, domestic swine (*Sus scrofa*) are considered a clinically important model for the study of factors affecting human gastrointestinal development, immunity and brain development [8,9]. Not only for this reason, but also due to its influence on physiological, nutritional and immunological processes, the structure and functional role of pig intestinal microorganisms has been an important subject of research for decades [10,11,12,13]. This relationship is an important issue for the global pork agribusiness.

The current review presents the current state of research on the pig digestive tract microbiome in order to better understand host-microbial interactions and their functions.

## 2. Development of Microbiota in Piglets 

It was believed for a long time that the gastrointestinal tract of animals was sterile before parturition, and its colonization with microorganisms began during birth from the mother (vertically—contact with vaginal microbiota, skin and feces of the mother), as well as from other individuals and the environment (horizontally) [14,15]. However, recent studies on both humans and animals suggest that microorganisms can also be found in the fetus, placenta, amniotic fluid or uterus [16,17,18,19], which indicates that microbial colonization has already started before parturition. This hypothesis was confirmed, for example, in the study conducted on cattle by Alipour et al. [20], who also confirmed that the microbiota of the gastrointestinal tract is subject to rapid changes after birth.

In general, the gastrointestinal tract of animals, including pigs, consists mainly of commensal bacteria and transition bacteria (including pathogens) interacting with each other [21]. Microorganisms inhabiting the gastrointestinal tract include the so-called indigenous bacteria, which permanently colonize the organism, and others, non-indigenous ones, that can only be observed temporarily [22]. Generally, the diversity of bacterial species and their abundance increase with animal age [23]. Initially, the digestive system of piglets is colonized by facultative aerobic or anaerobic bacteria. This composition is related to the colostrum and then milk, which contains mainly lactic acid bacteria, such as *Lactobacilli* and *Bifidobacterium*. Later, under the influence of diet and environmental factors, it is changed and finally stabilized [2,24,25,26]. This all suggests that gaining understanding related to the dynamics of piglet microbiota changes is an important issue, as microbiota can affect the health status and productivity of adult animals. 

Literature reports demonstrate that, after the birth, the gastrointestinal tract of piglets is mainly colonized by families of Clostridiaceae and Enterobacteriaceae [25,27,28,29]. The study by Swords et al. [27] showed that, 3 h after birth, the most abundant bacteria in the gastrointestinal tract were aerobic bacteria, followed by anaerobic ones and coliforms. Another study demonstrated that the *Streptococcaceae* family was observed in the gastrointestinal tract of piglets 6 h after birth, and they were the most abundant in the period from 1 to 3 days of life, when they were replaced by *Lactobacillaceae* and *Clostridiaceae* as a result of the so-called secondary colonization process [29] (Table 1). In turn, Konstantinov et al. [25] noted the presence of *Escherichia coli*, *Shigella flexneri*, *Lactobacillus sobrius*, *Lactobacillus reuteri* and *Lactobacillus acidophilus* in two-day-old piglets. The activity of E. coli and Clostridium spp. is observed within the first 6 h after the birth. However, the activity of Lactobacillus spp. is visible 24 h after parturition and Bacteroidetes spp. is the latest—four days after birth. The number of aerobic bacteria increased until about seven days after birth, then their number decreased significantly in favor of anaerobic bacteria [27]. In turn, according to Petri et al. [29], Lactobacillaceae were the most abundant group over a period of the first 20 days of life, which is in contrast to the results presented by Swords et al. [27] and Inoue et al. [28], who observed replacement of this group of microorganisms with Clostridium spp. However, there are significant changes in the microbiota during growth. A significant decrease in the level of Clostridium spp. and a significant increase in anaerobic bacteria count can be observed between 60 and 120 days of life. There is also a decrease in the number of Lactobacillus spp., which between 2 and 10 days of life constitute from 8% to 10% of the microorganisms found in feces, while during the growth and development of piglets their level drops below 1% at day 120. According to many authors [24,30,31], Lactobacilli constitute the predominant part of the bacterial community of the gastrointestinal tract of pigs, and are always present in it throughout an animal’s life. However, a significant increase in Bacteroides spp. count is observed between 30 and 120 days of piglets’ life, their population in this period is about 61% [27,32]. Some studies emphasize that the microbiological composition of piglets’ digestive tracts is quite stable during the suckling period, while the abundance of bacteria is subject to a rapid increase over that time [30,33,34]. The range of opinions presented in the literature mean that there is still a need to study issues related to microflora of the digestive tract from the first days of animal life.

After the weaning period, there are changes in the microbiota of the digestive system, which is stabilized after three consecutive months at the age of about 120 days [27]. During this time, the intestines contain mainly anaerobic, Gram-positive bacteria, and up to 10% of Gram-negative ones [35]. In this period, its composition is similar to that of adult individuals, where the main phyla, including Firmicutes, are: Clostridia spp.; Bacteroidetes, Proteobacteria, Spirochaetes and Synergistetes. However, their ratio varies from one section of the gastrointestinal tract to another. In the ileum, about 95% are bacteria from phylum Firmicutes and about 5% Proteobacteria. In the cecum, phylum Bacteroidetes constitute about 50%, Firmicutes about 40%, Proteobacteria about 5% and Spirochaetes about 4%. In the central part of the colon, the main phylum is again Firmicutes, constituting about 60%, followed by Bacteroidetes—30%, Proteobacteria, Spirochaetes, Synergistetes and others below about 10% [2].

## 3. Some Factors Affecting Piglet Microbiota Destabilization 

The changes occurring in the microbiota of the piglet digestive system are significantly affected by diet and environment [36,37,38,39]. Long-term stress increases the level of cortisol in piglets, which exacerbates the state of immunosuppression [40]. As a result, it induces disorders in secretion of saliva, gastric juice and digestive enzymes, which adversely affect intestinal peristalsis (mucositis, gastric and intestinal ulceration). Dietary and absorption disorders result in slower growth or weight loss [41]. One of the main stress factors in piglets, affecting the destabilization of the intestinal microbiota, is weaning at 28 days after birth [28,42,43,44]. This causes a decrease in the diversity and number of microbial populations in the gastrointestinal tract. During this period, the intestines of piglets are more vulnerable because of changes in the structure and barrier properties, which can lead to digestive disorders, diarrhea and growth retardation, as well as an increased mortality rate [2,45,46,47]. This confirms the importance of microorganisms inhabiting the gastrointestinal tract in young animals in terms of the maintenance of good health status.

In addition to stress, weaning is also a period of significant changes in the diet of piglets that can also result in changes to the microbiota inhabiting their gastrointestinal tract [48]. Zivkovic et al. [49] presented the concept of the so-called “milk-oriented microbiome” (MOM) which assumes that milk, especially glycans contained in it, can in some way control the microbiome of nursing animals. This was confirmed in the study by Frese et al. [50] who analyzed the fecal microbiome of pigs for the first seven weeks of their life. During this time, the diet of piglets changed from sow milk only to a diet including components of plant and animal origin. The authors concluded that the intestinal microbiome of piglets was to a great extent affected by dietary glycans, which was reflected in different functional possibilities of this microbiome in periods before and after weaning [50]. Thus, diet can be a factor allowing a certain extent of control over the composition of digestive system microorganisms. 

Those pathogenic bacteria the multiplication of which most often causes intestinal disorders include Escherichia coli, Clostridium perfringens, Salmonella choleraesuis and Salmonella typhimurium. They produce enterotoxins which may cause rhinitis, and damage and even complete impairment of the intestinal villi. As a result of bacterial infection, the permeability of fluids to the intestinal lumen leading to diarrhea increases. In addition, an increase in pH hinders the multiplication of beneficial lactic acid bacteria, leading to a worsening of the disease [51]. Post-weaning diarrhea is characterized by a decrease in the number of commensal bacteria, including Lactobacillus sobrius, L. acidophilus and L. reuteri, and an increase in pathogenic E. coli count [25,52,53]. By damaging the intestinal villi, the microorganisms accelerate the division of cells in the epithelium of the small intestine which leads to its peeling. The cells cannot reach full maturity, so they do not perform their basic functions and do not participate in digestive processes. Therefore, piglets are not able to digest carbohydrates that pass into the large intestine (the microbiota of the digestive system is not fully developed, unable to effect bacterial digestion) [54]. 

## 4. Probiotics and Prebiotics as an Alternative to Antibiotics Use

In the last 50 years, in order to remedy the problem of weaning diarrhea, antibiotic treatments have been used that substantially alter the structure of the intestinal microbiota in piglets. In addition, such a policy has led to the emergence of strains of pathogenic bacteria resistant to antibiotic therapy and has created a threat to the health of both animals and humans [30,45,55,56,57].

According to the latest research, the main and most satisfactory alternatives to antibiotics are probiotics and prebiotics, which can replace the antibiotics in the feed [30].

Probiotics are living organisms that can bring health benefits to the host when administered at the right time and in the right amounts [58]. The main microorganisms exhibiting probiotic activity are bacteria of the genera Lactobacillus, Bifidobacterium, Gram-positive bacteria of the genera Streptococcus, Enterococcus, Pediococcus, Lactococcus, Leuconostoc, Bacillus and Propionibacterium (P. freundenreichii), yeasts of the genera Sacharomyces, Kluyveromyces, and fungi of the genus Aspergillus [59,60,61]. 

Collins and Gibson [62] concluded that an effective probiotic should demonstrate a profitable effect on a host’s organism, should not be pathogenic or toxic, should contain a large count of viable cells and be able to survive and metabolize in intestines, should be able to survive storage and use periods and be characterized by good sensory traits, and finally should be isolated from the same species as the host it is intended for [62]. In addition, probiotics provide the possibility to manipulate the microbiome, especially in young animals, in order to achieve better animal health, welfare and productivity. Probiotics cause a decrease in pH to acidic in the digestive system, compete with pathogens for nutrients and adhesion to intestinal epithelial receptors, and release antimicrobial and toxin inactivating substances [54,63,64,65]. They influence the stabilization of intestinal microbiota, increase the absorption surface (growth of intestinal villi), improve the digestibility of dry matter, lead to better use of feed and, above all, reduce the incidence of diarrhea in piglets [66,67].

Probiotic bacteria should be characterized by an established taxonomic affiliation, adhesion to the epithelium of the intestine (in order to quickly multiply and colonize the tract and reduce the adhesion capacity of pathogenic bacteria), they should reduce availability of nutrients to pathogenic organisms, have the ability to lower the pH of the intestinal environment, be characterized by high enzymatic activity, and synthesize certain vitamins (B, PP, K). Probiotic bacteria stimulate immune mechanisms, mucus production and show bacteriostatic and bactericidal activity, but they cannot produce substances harmful to animals or show side effects [68,69,70,71].

It is also known that the use of a probiotic is specific to the treatment, depending on the specific strain, dose and context, as well as the specificity of the host, in particular its physiological parameters (e.g., health status and genetics) or environment ( e.g., sanitary status and diet) [66,72]. Studies have also demonstrated that probiotics can modulate immune functions in the host [73,74]. Strains should be selected according to the objectives and the use should be targeted specifically at the effect (Table 2). Many literature reports have demonstrated that administration of probiotics to pigs results in an improvement in production parameters such as daily gains, feed intake or feed conversion ratio [54,67].

The summary presented in the review paper by Turner et al. [77] allows the conclusion that most probiotics used in the studies presented in the paper had a positive effect mostly on growth rate, then feed efficiency and gut function, while the least remarkable results were found in the case of immune function and feed intake [77]. A positive effect of the probiotic on the health and composition of intestinal microorganisms in piglets was proven, for example, in a study carried out using Lactiferm AD3EFe++ probiotic paste, containing Enterococcus faecium strain from days 3 to 48 of piglets’ life. Mortality in the control group was 13%, while in the experimental group it was 0%. However, the growth of piglets in the experimental group was significantly lower. The authors point to two possible causes of reduced growth rate in the experimental group: excessive litter size compared to the control group and a dosage of probiotic other than that recommended by the manufacturer [78]. In the study conducted by Scharek et al. [79], determining the effect of an administration of probiotics with Enterococcus faecium strain to pregnant sows and piglets, no clear improvement in immune system stimulation was demonstrated, but an effect on the development of beneficial microbiota of the piglets’ digestive system was noted. Another positive probiotic effect on health was presented in the study by Bohmer et al. [80] who used Enterococcus faecium DSM 7134. A significant piglet mortality rate (about 2%) was eliminated and piglets in the group of sows supplemented with probiotic were characterized by a higher body weight compared to the control group. The use of probiotics also increased the sows’ resistance to pathogens. The study suggests a significant influence of probiotics on the health status of sows and the number of piglets born. The results showed that, despite the already stable microbiome in adult animals, disturbance may occur, which results in a decrease in production capacity. 

An increase in the number of E. coli in the intestines is a quite common problem in the case of pigs. Studies carried out using probiotics have demonstrated their effect on E. coli. In the case of pigs treated with antibiotics, the level of E. coli in the intestines dropped dramatically after four weeks, which was not shown in untreated pigs. In addition, some probiotics improve the quality of colostrum and milk in sows (such genera as Bacillus, Lactobacillus and Streptococcus), which results in better weight gains and improved immunity and health status of piglets [54,59]. It should also be emphasized that probiotics in pigs mainly affect the colon and cecum, which are inhabited by abundant and diverse populations of microorganisms [54].

Prebiotics represent another approach to host protection against pathogen infections. The idea of prebiotics assumes a dietary route to the improvement of beneficial microbiota status in the gastrointestinal tract, and mainly non-digestible oligosaccharides such as fructooligosaccharides (FOS), mannanoligosaccharides (MOS), galacto-oligosaccharides (GOS), and xyloligosaccharides (XOS) are used for this purpose [81,82,83,84]. These are natural ingredients of various plants and, in lower amounts, also cereals. The task of these compounds is to selectively stimulate the growth or activity of some bacteria such as Lactobacilli or Bifidobacteria and counteract the growth of pathogenic bacteria, which is beneficial from the host’s point of view. A combination of a probiotic and a prebiotic is called a symbiotic [30,59,81,85,86]. However, as the majority of prebiotic effects, such as diarrhea or constipation prevention or intestinal microbiota metabolism modulation, are indirect, which means they are mediated by microorganisms colonizing the digestive tract, they are also less-well proven [87]. This was emphasized by Jacela et al. [64] who also concluded that more studies are needed to prove definitively any positive influence of prebiotics on the pig organism.

## 5. Effect of Diet on Swine Microbiota and its Influence on Production Indices

Microbiological homeostasis of the digestive tract ensures proper functioning of an animal’s organism. Proper microbial activity adapted to the host ensures proper nutrient utilization and proper growth and development. Both enzymatic hydrolysis and microbiological decomposition of nutrients are important for the digestion of nutrients in pigs [88,89].

Microorganisms inhabiting the gastrointestinal tract contribute to the production of vitamins and cofactors that decompose previously indigestible feed components, and bacteriostatic and antifungal substances, thus reducing populations of pathogenic microorganisms. It is important in animal production to maintain microbial homeostasis using various strategies, such as optimizing animal diets and ensuring appropriate zoohygienic conditions. Suitable microbiota in the pig gastrointestinal tract can ensure increased health status and thus correct or increased production indices [11,54,90,91,92]. 

Diet and its additives are the most controlled factors influencing the microbiological composition of the digestive system. Changes in the microbiota may be caused by an increase in fiber content, as this is quite sensitive to its level in the feed ration of pigs [89]. Then, xylanolytic and cellulolytic bacteria count is subject to an increase [93,94]. Changes also occur when zinc oxide (ZnO) is added to pig feed to prevent diarrhea. After analysis of the microbiome of pigs, changes at the genus level have been found. The number of bacteria of the genera Weissella, Leuconostoc, Streptococcus increase, while the number of Sarcina decrease significantly [95]. As shown in these studies, component modifications in the diet cause changes in the microbiota of the gastrointestinal tract of pigs, which adapts to these changes in order to survive [58]. A study carried out by Leser et al. [35] showed the influence of diet on the improvement of the health condition of pigs. A diet based on boiled white rice used in the study reduced the incidence of dysentery in pigs; however, increasing the amount of dietary fiber above 20% resulted in an increase in the count of Brachyspira hyodysenteriae and an increase in the risk of dysentery. Positive effects were also observed when the diet was fermented and supplemented with an organic acid, i.e., lactic acid [35].

In the case of pigs, stress is another factor that significantly affects the microbiological composition of the digestive system. This may be caused by many factors, which include weaning, transport and feed reduction [94,96]. In the case of weaning, the number of Lactobacillus increases and Bifidobacterium and E. coli decrease. The dominant bacteria are L. sobrius and L. amylovorus. The relative stability of microbiota starts around 11 days after weaning [97]. In the case of transport stress, however, the population of Salmonella typhimurium in fecal samples of pigs hosting these bacteria is subject to an increase. A significant level of these bacteria was found in pigs fed before transport to the slaughterhouse, but it was not demonstrated when feed was withdrawn 24 h before slaughter [98].

The development and subsequent stability of gastrointestinal microorganisms is essential for the normal dietary, physiological and immunological functions of pigs. Disturbances in the intestinal microbiome create an opportunity for pathogenic microorganism development which may result in increased disease occurrence. Common management practices in intensive pig production, such as early and sudden weaning, poor hygiene, and prophylactic use of antibiotics, can cause disturbances in the intestinal microbial ecosystem, exposing animals to the development of pathogens and consequently diseases [52,59,99].

The microbiota of the digestive system not only play a protective role, but can also influence the production parameters of pigs. Changes in the microbiota of the digestive system affect assimilation of nutrients, and in the case of fattener pigs, also the quality and quantity of meat [4,91]. The study by Park et al. [4] showed changes in the composition of microorganisms at the genus level between individuals characterized by high meat quality and those with low meat quality. The study showed slightly higher levels of Lactobacillus and Oscillibacter in individuals with high quality meat compared to low quality ones. Higher levels of Roseburia spp. and Clostridium spp. were also found in animals with higher meat quality. Additionally, some strains such as Roseburia intestinalis L1-952 may produce conjugated linoleic acid (CLA) from linoleic acid (LA), which may reduce the amount of fat tissue [4,100]. The acetate produced by some Clostridium strains, such as Clostridium mayombei, is a component of short-chain fatty acids (SCFA), the function of which is, among others, to supply the body with energy [101,102]. The studies by Park et al. [4] and Devillard et al. [100] suggested that CLA and SCFA decomposition by Clostridium spp. may have a positive effect on the quality of meat in pigs, as well as on the composition and amount of fat in meat.

The microbiota of the digestive system of pigs also fulfil protective functions and ensure, apart from the quality of meat, an appropriate rate of animal growth. As described above, the composition of the microorganisms may significantly affect the quality of meat, but if it is disturbed by external factors, production parameters such as weight gain may be reduced [6,10]. Changes in the composition and abundance of microorganisms may increase the risk of disease. Studies on immune response to PRRSV and porcine circovirus-2 virus (PCV 2) have shown that the microbiota—its abundance and diversity—influence the level of animal morbidity, as the virus makes secondary infection more probable. Both viruses impede host defense mechanisms and increase susceptibility to primary and secondary infections with pathogens that may affect growth rates, morbidity and mortality [6]. It was demonstrated in the study by Niederwerder et al. [6] that infections with PVC2 and PRRSV significantly reduced animal growth in groups of individuals characterized by less diverse microbiota and in groups with poorer clinical results, in which the weight gains over a period of 70 days were 0.36 kg vs. 6.91 kg. It was also shown that in the group with the best clinical results the occurrence of phylum Proteobacteria bacteria was observed (E. coli, E. amylovora, C. lari, D. suillum, M. hematolytica), which were not found in the group with the worst clinical results. Additionally, the study by Jaing et al. [103] also showed a correlation between the occurrence of PRRSV and opportunistic bacteria such as M. hyopneumoniae (identified in serum), P. multocida, A. pleuropneumoniae and S. suis associated with the respiratory system of pigs. These studies also showed that the PRRSV virus increased susceptibility to lung disease in pigs caused by S. suis.

Changes in the composition and abundance of microorganisms inhabiting pigs’ digestive tracts may also affect some reproduction parameters. High resistance to external pathogens is important in the case of sows during parturition. Microbiota disturbance in the perinatal and postpartum period in sows may cause "starvation infertility", resulting in a lower number of piglets. This is mainly caused by deficiencies in nutrient intake related to the lactation period and weaning of piglets. A decrease in feed intake and a high mobilization of body tissue combined with a lack of energy can be observed during lactation [76,104]. In such cases, studies have shown significant improvements in terms of both the number of piglets and reduced mortality resulting from probiotics and the sow’s diet.

The results of the studies conducted demonstrated the importance of the microbiome of the digestive system of pigs and its influence on production indices. Interestingly, the differences in the composition and abundance of the microorganisms have allowed the demonstration of the interdependence of the level of some groups of bacteria (e.g., M. hyopneumoniae, P. multocida, A. pleuropneumoniae and S. suis) and disease occurrence or a decrease in production indices. Therefore, further study into the relationship between factors lowering production indices and the microbiome should be undertaken.

## 6. Conclusions

Studies into the microbiome and its functions in pigs enable a thorough understanding of its importance for the host. The mutual relationships between the microorganisms allow the maintenance of an appropriate level of animal resistance to pathogens, but also affect production indices. Investigation of the efforts of pig breeders aimed at providing stable microbiota can bring measurable benefits. The use of preparations supporting the development and colonization of microbiota, such as prebiotics and probiotics, from the very beginning of an animal’s life may prevent the risk of multiplication of pathogens causing, for example, diarrhea. Then, the use of a diet with additives, such as boiled rice or lactic acid, may reduce the incidence of not only digestive system diseases, but also systemic diseases, due to the action of the microbiome stimulating the specific immune system of the host.

## Figures and Tables

**Table 1:** Examples of bacteria detected in various time periods after the birth [25,26,27,28,29].

Time after Birth	Detected Bacteria
3 h	*Clostridiaceae, Enterobacteriaceae*
6 h	*Streptococcaceae*, *Escherichia coli*, *Clostridium* spp.
1st–3rd day	*Lactobacillaceae* (*L*. *sobrius*, *L*. *reuteri*, *L*. *acidophilus*), *Bacteroidetes* spp., *Escherichia coli, Shigella flexneri*

**Table 2:** Examples of bacteria used as probiotics in piglet nutrition [54,63,66,75,76].

Bacteria	Effects on Piglets
*Bifidobacterium lactis* HN019	Reduced diarrhea, better productivity
*Bacillus subtilis*	Weight increase
*Lactobacillus rhamnosus* ACTT 7469	Reduced diarrhea, lower level of *E. coli* in feces
*Bacillus licheniformis*	Reduced diarrhea
*Lactobacillus murinus* DPC6002 and DPC6003	Reduced diarrhea, better productivity
*Enterococcus faecium* DSM 7134	Better growth of piglets, higher resistance to pathogens, lower mortality
*Bacillus*, *Lactobacillus* and *Streptococcus*	Improved quality of colostrum and milk
